# Correlation between antibiotic resistance and phylogenetic types among multidrug-resistant *Escherichia coli* isolated from urinary tract infections

**DOI:** 10.22038/ijbms.2021.47095.10865

**Published:** 2021-03

**Authors:** Humera Nazir, Mubashar Aziz, Zulfiqar Ali Mirani, Ahsan Sattar Sheikh, Muhammad Qamar Saeed, Aleem Ahmed Khan, Tahira Ruby, Naseem Rauf

**Affiliations:** 1 Department of Microbiology, Quaid-i-Azam University, Islamabad, Pakistan; 2 Institute of Pure and Applied Biology, (Microbiology division) Bahauddin Zakariya University, Multan 60900, Pakistan; 3 Department of Microbiology, PCSIR Laboratories Complex, Karachi 74000, Pakistan; 4 Institute of Molecular Biology & Biotechnology, University of Lahore, Lahore, Pakistan; 5 Department of Zoology, The Islamia University of Bbahawalpur,61300, Bahawalpur, Pakistan; 6 PCSIR Laboratories Islamabad, Pakistan

**Keywords:** Escherichia coli, ESBL, MDR, PCR, UTI

## Abstract

**Objective(s):**

Emergence of multidrug resistance has reduced the choice of antimicrobial regimens for UTIs. To understand the association of phenotype and genotype among uropathogens.

**Materials and Methods:**

Six hundred and twenty-eight (628) urine samples were collected and analyzed. Antibiotic sensitivity pattern was determined by the Kirby-Bauer Disc Diffusion Method and minimum inhibitory concentration (MIC) was tested by the E test. Fluoroquinolone resistant mutations in QRDR of *gyrA* and *ParC*, phylogenetic groups, and PAI*usp* subtype were detected by PCR.

**Results:**

Most prevalent uropathogens were *Escherichia coli* (53.2%) followed by *Klebsiella* *pneumoniae* (21%). Multidrug- resistance was observed in > 50% cases for third-generation cephalosporins and ciprofloxacin and lowest in meropenem. *E. coli *(66.2%) and *K. pneumonia *(64.4%) were extended-spectrum β-lactamases (ESBLs) producers. MIC to trimethoprim-sulfamethoxazole was highest in *E. coli* (>1024 µg/ml). In 80 (24%) of the 334 *E. coli *isolates analyzed in detail, 54 fluoroquinolones (FQ) resistant isolates carried mutations (S83L, D87N, S80I, E84V) in QRDR of *gyrA* and *ParC*. Out of 54 FQ-resistant isolates, 43 (79.6%) isolates belonged to the phylogenetic group B2, and 11(20.4%) belonged to group D. Isolates belonged to group B2, 38 (88.4%) of the 43 isolates carried PAI*usp* subtype IIa and high frequency of mutation E84V in *ParC* was detected in 37 (97.4%). Other mutations, such as S80I, S83L in *gyrA* and D87N in *ParC* were found in all resistant isolates.

**Conclusion:**

Correlations between phenotype and genotype provided a basis to understand the resistance development in uropathogens, and PAI*usp *subtyping indicated that *E. coli* belonged to the B2 group.

## Introduction

Urinary tract infection (UTI) is among the most common infectious diseases in both community and hospital settings. UTIs are caused by multidrug-resistant bacteria and serves as a therapeutic challenge for general family practitioners and hospital doctors in developing countries (1, 2). UTIs, if diagnosed early, and with adequate antibiotic coverage, are not alarming. However, if inadequately treated, these infections can cause significant morbidity and mortality (1,2). UTIs can be classified as community-acquired (CA) or hospital-acquired (HA). About 70 to 80% of CA-UTI is caused by multidrug-resistant *Escherichia coli *from the patient’s normal gut flora. *E. coli *isolates that cause UTI and may cause extra-intestinal infections are responsible for epidemic spread within the population (3). In several studies, Gram-negative bacteria are responsible for 80–85% of UTIs and major causative organisms are *E. coli *(75–87%) followed by *Klebsiella *spp.* Citrobacter *spp*, Enterobacter *spp*, Pseudomonas *and* Proteus *spp (4, 5). Women are more prone to UTIs than males. Higher incidence in females is due to urothelial mucosa adherence to mucopolysaccharide lining (6). In old age, the chance of UTI increases due to menopause (estrogen loss) in females. Furthermore, prolonged use of antibiotics can also damage periureteral flora resulting in more colonization of uropathogens to the urinary tract (7). UTIs are commonly treatable with antibiotics and the selection of empirical therapy should be based on the prevalent antibiogram data (8). The irrational use of antibiotics without proper antibiotic susceptibility testing has led to an extensive increase in drug-resistant UTIs pathogens that have made treatment more difficult. Overall, this situation has led to adverse consequences like high morbidity and mortality, prolonged hospital stays with higher costs and increased antimicrobial resistance, and a surge in nosocomial infections (9- 11). The most commonly used antibiotics for routine UTIs are ß-lactam (penicillins, cephalosporins, and carbapenem), followed by fluoroquinolones, trimethoprim, trimethoprim/sulfamethoxazole, fosfomycin, nitrofurans and aminoglycosides (12). Fluoroquinolones are widely prescribed as empirical therapy in Europe and the USA (11, 13). Acquisition of high-level resistance involves several factors and in the majority of cases, resistance arises spontaneously due to point mutation within g*yrA, gyrB, parC, *and *pares *genes (14). Qnr genes produce proteins that can bind to DNA gyrase, protecting it from the action of quinolones. Ultimately, the drugs effectively can be reduced with mutations at main sites in DNA gyrase or Topoisomerase IV, which can cause a decrease in their binding affinity to quinolones (15-17). Resistance to ß-lactam antibiotics among clinical isolates of *E. coli *and *K. pneumoniae *is most often due to the production of ß-lactamases (18, 19). These enzymes confer resistance to extended-spectrum (third-generation) cephalosporins and monobactams (e.g., aztreonam). ESBLs genes are located on large ESBL plasmids which also confer resistance to other antibiotics including aminoglycosides, chloramphenicol, and sulfonamides and limiting their empirical and rational use for clinicians (20, 21). Phylogenetic grouping of *E. coli *isolates is a more convenient and faster triplex PCR method as previously reported (16). This categorizes *E. coli *isolates into four major phylogenetic groups A, B1, B2, and D. The PAI *usp *(putative pathogenicity island carrying uropathogenic-specific protein) subtypes, Ia, Ib, IIa, and IIb were also determined according to their DNA sequence in *usp *variants, *uspI *and *uspII *(17, 21). The present study aimed to investigate the prevalence of uropathogens among patients admitted to tertiary care hospitals in Islamabad, Pakistan, and to study the antimicrobial resistance patterns of ESBLs and non ESBLs isolates to different classes of antibiotics. Further analysis was done to understand the distribution and correlation among phylogenetic groups, PAI*usp *subtype, *gyr A *and *parC *genes in uropathogenic *E. coli.*

## Materials and Methods

***Isolation and identification of uropathogens***

Urine samples were collected from patients with UTI or suspected UTI who visited OPD or were admitted to one of three different hospitals in Islamabad, Pakistan: Pakistan Institute of Medical Sciences (PIMS), the Government Services Hospital, and the Capital Development Authority (CDA) Hospital Islamabad. A total of 628 urine samples were examined and cultured on Cystine Lactose Electrolyte-Deficient (CLED) agar as per standard microbiological protocols.

***Inclusion and exclusion criteria***

 Patients’ urine cultures yielding a pure growth of ≥105 CFU/ml were considered significant and isolates were further processed for identification and susceptibility. However, the patient’s urine yielding a count of ≤105 CFU/ml was considered insignificant and was excluded from the study (21). Patients’ demographic data were obtained from computerized laboratory reports.

***Susceptibility testing and minimum inhibitory concentrations (MIC)***

Antibiotic susceptibility testing was determined using the Kirby-Bauer disc diffusion method (22) as per CLSI guidelines to classify isolates as susceptible, intermediate, or resistant (23). The antimicrobial agents used were ampicillin (AMP), ceftazidime (CAZ), cefotaxime (CTX), cefuroxime (CXM), amoxicillin-clavulanic acid (AMC), meropenem (MEM), nitrofurantoin (NIT), amikacin (AMK), chloramphenicol (CHL), trimethoprim (TMP), and ciprofloxacin (CIP). Minimum inhibitory concentrations (MIC) were determined by the broth microdilution method (23). *E. coli *(ATCC 25922) and *K. pneumoniae *(ATCC 33945) were used as quality control isolates.

***Phenotypic detection of ESBLs***

Screening for ESBLs was performed by both double-disc diffusion and Broth Microdilution Assays, as reported previously (24). Briefly, in the Disc Diffusion Assay BD Sensi-discs (Becton Dickinson, USA) were used with the following concentrations; cefotaxime (CTX) 30 µg; cefotaxime 30 µg plus 10 µg clavulanate (CA); ceftazidime (CAZ) 30 µg; and ceftazidime 30 µg plus 10 µg clavulanate (CA). A test was considered positive for ESBLs when the zone for cefotaxime (CTX) was 27 mm, and the zone for ceftazidime (CAZ) was 22 mm, and if the diameter of zones of inhibition was ≥5 mm increased in the zone of inhibition for the CAZ/CA and CTX/CA-containing discs versus the corresponding CAZ or CTX disc. Broth microdilution test was considered positive if MIC for either drug alone was 2 mg/l and if there was a decrease of at least 3 twofold dilutions for the combinations with clavulanate.

***PCR amplification and sequencing***

*gyrA *and *parC *genes were identified and amplified by PCR using primers described previously (25). PCR experiments were carried out using PTC-200 Peltier thermal cycler (SDS Diagnostics, Sweden) with primers from Sigma (Sigma Genosys Ltd, Sigma-Aldrich House, UK) according to the following conditions: denaturation at 95 °C for 5 min, 30 cycles of 94 °C for 15 sec, 53 °C for 20 sec, and extension at 72 °C for 1 min. PCR products were visualized by agarose gel electrophoresis. Purification was done before DNA sequencing using a QIA quick PCR purification kit (Qiagen, VWR International AB, Stockholm, Sweden) and quantified using a NO-1000 spectrophotometer (NanoDrop Technologies, USA). PCR products were sequenced at Macrogen Inc., Seoul, South Korea.

***Phylogenetic grouping of uropathogenic E. coli***

The phylogenetic group was determined by the triplex- PCR- based method reported previously (26). The PCR based method uses three primer pairs ChuA-1, ChuA-2, YjaA-1, YjaA-2 and TSPE4C2.1, TSPE4C2.2 to amplify the bacterial DNA, which generated fragment sizes of 279, 211, and 152 bp, respectively. *E. coli *isolates were classified into phylogenetic groups A, B1, B2, and D by using the results of PCR amplification (Table 1). Subtypes of PAI *usp* were recorded by the PCR technique as reported previously (17). PAI *usp *variants were classified into four subtypes, Ia, Ib, IIa, and IIb according to the DNA sequence of the *usp *variants.

## Results

A total of 628 urinary isolates were collected from both in-patient and out-patient departments over 10 months with a significant culture count of ≥105 CFU/ml. Of 628 uropathogens isolated, 466 (74%) accounted for two species with high frequencies: *E. coli *334 (53.2%) and *K. pneumoniae *132 (21%). Species found at lower frequencies included: *Enterobacter *(11.6%), *Proteus *spp. (6.5%), *P. aeruginosa *(4.6%), and *S. aureus *(3.0%). *E. coli *and *K. pneumoniae *(74%) were considered for further analysis of susceptibility and resistance patterns. The vast majority of isolates were community-acquired 402(86%) followed by hospital-acquired isolates 64 (14%). Out of 402 community-acquired UTI isolates *E. coli *was predominant in 68.7% (276/402) followed by *K. pneumoniae *31.3% (126/402). Among 64 cases of nosocomial UTIs, 51(79.6%) were caused by *E. coli *and 13(20.3%) by *K. pneumoniae*. The gender of the patients in UTIs showed that 264 (57%) were females and the majority were infected with *E. coli *192 (72.72%), whereas 72 (27.3%) were infected with *K. pneumoniae. *Among 202 (43%) male patients, 70.3% were infected with *E. coli*, whereas *K. pneumoniae *was the causative agent in 60 (29.7%) cases. Approximately two-thirds of the uropathogens were ESBL producers (Table 2). Highest percentages of *E. coli *(n=94, 28.14%) and *K. pneumoniae *(n=36, 27.27%) were isolated in elderly people (51–60 years) followed by (41–50 years group) having *E. coli *(n=75, 22.45%) and *K. pneumoniae *(n=29, 21.96%). The lowest prevalence was among patients having age <20 years. The prevalence of UTIs was found to be higher in the period from June to November. Among 466 cases, the highest percentage of UTIs, 68 (n=68, 15.2%), was observed during July 2016, while the lowest percentage of these infections (n=22, 6.58%) and (n=24, 5.6%) was found during February and March 2017, respectively.

A total of 466 uropathogens including *E. coli *and *K. pneumoniae *were screened for their drug susceptibility patterns by the disc-diffusion method. Out of 334 isolates tested, *E. coli *exhibited the highest resistance against ampicillin (87%). However, a relatively lower bacterial resistance (51%) was observed against β-lactam/β-lactamase-inhibitor combination such as amoxicillin-clavulanic acid. Among cephalosporins, *E. coli *had a higher resistance (77%) against first-generation antibiotic cefazolin, while resistance against second and third-generation cephalosporins was recorded as 75% and 69%, respectively. In aminoglycosides, amikacin (35%) showed better antibacterial activity than gentamicin (50%). Bacterial resistance was also higher against quinolone and other tested drugs. In the case of *K. pneumoniae*, the highest rate of resistance was observed against ampicillin (100%) and the lowest against meropenem (21%). Resistant isolates were also in higher proportion against various tested drugs.

***Susceptibility pattern of E. coli isolates***

**Figure 1:**
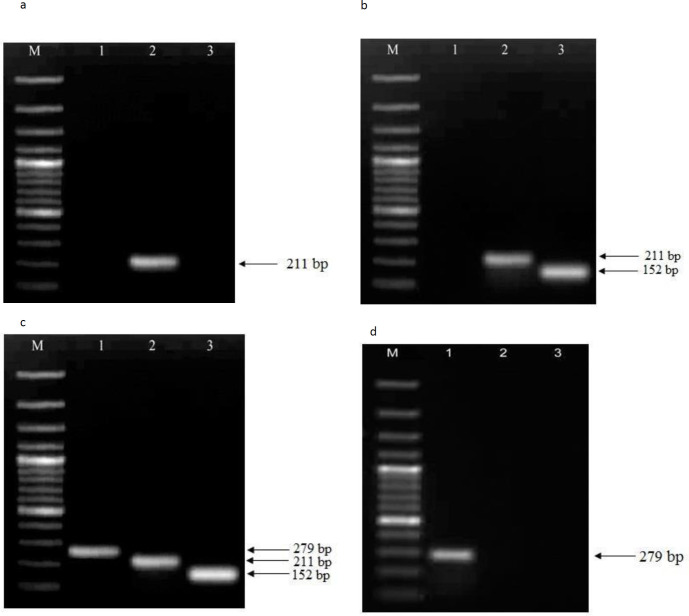
PCR profile specific for *Escherichia coli * phylogenetic groups. **a: **Each combination of ChuA, yjaA, and DNA fragment TspE4.C2 amplification allowed phylogenetic group determination of a strain. Lane M: 100 bp ladder, lane 1 chuA(-), lane 2 Yja A(+) and lane 3 TspE4.C2(-): Phylogenetic group A. **b:** Lane M: 100 bp ladder, lane 1 chuA(-), lane 2 Yja A(+) and lane 3 TspE4.C2(+): Phylogenetic group B1. **c:** Lane M: 100 bp ladder, lane 1 chuA(+), lane 2 Yja A(+) and lane 3 TspE4.C2(+): Phylogenetic group B2. **d:** Lane M: 100 bp ladder, lane 1 chuA(+), lane 2 Yja A(-) and lane 3 TspE4.C2 (-): Phylogenetic group D

MIC values were determined for resistant isolates of *E. coli *and *K. pneumoniae *against 5 antibiotics. For AMC, 9.36% isolates showed inhibition of growth at an antibiotic concentration of 16 µg/ml, 13.79% at 32 µg/ml, 16.25% at 64 µg/ml, 19.70 % at 128 µg/ml, 18.72 % at 256 µg/ml, and 22.2 % at 512 µg/ml (Figure 1). In case of CIP, most of the isolates (22%) showed inhibition of growth at 256 µg/ml concentration of antibiotics. 16.0% were inhibited at 128 µg/ml followed by 12.11% at 64 µg/ml, 9.34 % at 32 µg/ml, 8.59% at 512 µg/ml, while 3.90 % at concentration 1024 µg/ml.

In chloramphenicol-resistant clinical isolates, 38.70 % showed inhibition of growth at an antibiotic concentration of 64 µg/ml, 11.8% at 16 and 128 µg/ml. Low percentage of isolates (6.98%) showed MIC of 1024 µg/ml. TMP, exhibited MIC range 64 to >1024 µg/ml. Maximum isolates of *E. coli *23.54% were inhibited at >1024 µg/ml, whereas, 9.21% isolates were inhibited at 64 µg/ml. In case of SXT, relatively few of the isolates 3.98% showed inhibition of growth at 16 µg/ml. 6.88% showed inhibition of growth at antibiotic concentrations of 32 µg/ml, 8.33 % at 64 µg/ml, 11.59% at 128 µg/ml, 26.81% at 256 µg/ml, 13.04% at 512 µg/ml, and 13.76% at >1024µg/ml.

***Susceptibility pattern of K. pneumoniae isolates***

Amoxicillin-clavulanic acid (AMC), exhibited MIC range 16 to 512 µg/ml. Maximum isolates of *K. pneumoniae were *inhibited at 512 µg/ml (25.51%). Among AMC-resistant clinical isolates, 11.22% showed inhibition of growth at an antibiotic concentration of 16 µg/ml, 14.28% at 32 µg/ml, 17.34% at 64 µg/ml and 15.31% at 128 µg/ml, whereas 16.32% at concentration of 256 µg/ml. 

Maximum clinical isolates of *K. pneumoniae *for CIP (24.34%) were inhibited at 64 µg/ml and only 2% were inhibited at 1024 µg/ml and MIC range was 16 to 1024 µg/ml. In the case of CHL, a few of the isolates 7.3% showed inhibition of growth at 512 and1024 µg/ml, while the growth of the rest of the isolates was stopped at concentrations of antibiotics less than 512 µg/ml. For TMP, most of the resistant isolates (25.21%) showed inhibition of growth at >1024 µg/ml concentration of antibiotics. These clinical isolates of *K. pneumoniae *showed MIC range of 64 to >1024 µg/ml. Trimethoprim-sulfamethoxazole (SXT) exhibited the broadest range of 16 to 1024 µg/ml. 

Approximately, 18.01% isolates showed MIC level at 1024 µg/ml, followed by 16.21% at 256 µg/ml, and 15.31% at 64 µg/ml. Eighty uropathogenic *E. coli *were chosen randomly from the collection for more detailed genetic analysis. Of these 80 *E. coli *isolates, 54 were resistant and 26 were susceptible to ciprofloxacin according to EUCAST breakpoints, 2010.

**Figure 2:**
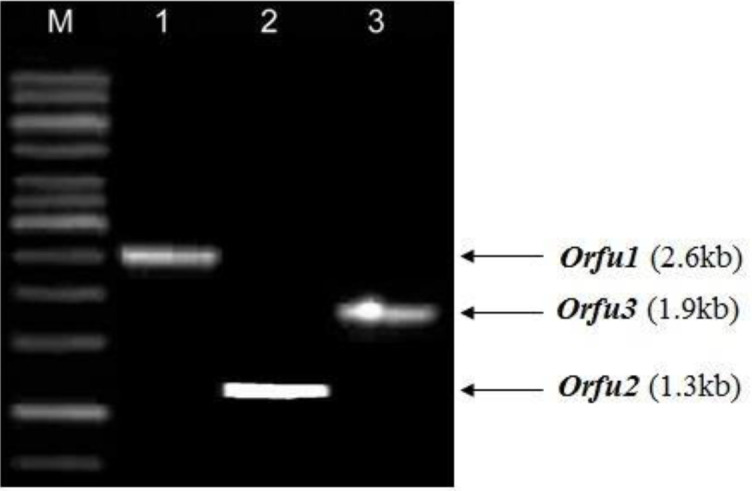
Profile after 1% agarose gel electrophoresis of the PCR product amplified by three sets of primers for subtyping of PAI *usp*. M: 1 kb ladder, lanes 1, 2, and 3 contain PCR amplicons by pairs of primer USP81f andORFU1r, USP81f, and ORFU2r, USP81f and ORFU3: Type IIa

**Figure 3:**
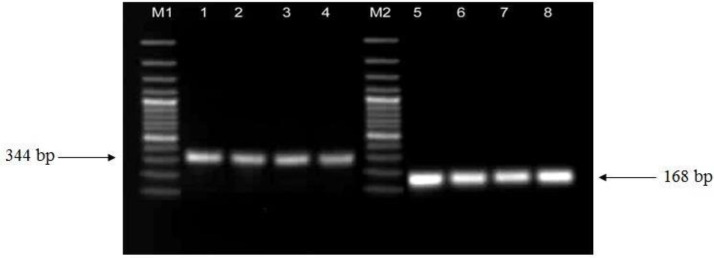
PCR amplification of *gyrA* and *par C *genes, lane M1, M2: 100 bp ladder, lanes 1-4: *Escherichia *coli clinical isolates (*gyrA*) and lanes 5-8 (*par C*)

The overall distribution of phylogenetic groups among 80 *E. coli *isolates was: A (7), B1 (11), B2 (48), and D (14), while phylogenetic group classification among susceptible isolates was A (7), B1 (11), B2 (5), and D (3) Figure 1 (a, b, c, d). Among the 54 FQ-resistant isolates, 43 (79.61%) isolates belonged to phylogenetic group B2 and 11(20.37%) isolates belonged to group D. None belonged to A or B1 groups. Isolates of *E. coli* presented positive results for *gyrA, par C* and PAI *usp* subtyping (Figure 2, 3). Distribution of the variations of PAI*usp* subtypes among FQ-resistant isolates is summarized in Table 2. Among the 43 FQ-resistant isolates belonging to the B2 group, 38 (88.37%) isolates were PAI*usp* subtype IIa. In contrast, none of the 11 FQ-resistant isolates carried PAI*usp* subtype IIa, which belonged to group D. Among the 11FQ-resistant isolates belonging to group D, 9 (81.81%) isolates were PAI*usp*- negative and two isolates carried PAI*usp* Ib. FQ- resistant isolates carrying resistance mutations in *gyrA* and *parC* are summarized in Table 5. Results showed 37 (97.36%) of the 38 isolates of PAI*usp* subtype IIa in phylogenetic group B2 carried E84V mutation in *parC*, while only two (18.18%) of the 11 isolates belonging to group D carried this mutation. In contrast, the mutations *parC* S80I, S83L, and D87N in *gyrA* were found in all of the resistant isolates (Table 2).

## Discussion

In our study, the most commonly isolated organism in UTI was *E. coli *(53.2%) followed by *K. pneumoniae *(21%) and *Enterobacter *(11.6%). These results are per findings of previous studies (2, 4, 8, 20, 26). Furthermore, most of the UTIs were observed to be community-acquired (86%) and the frequency of *E. coli *was more than that of *K. pneumoniae *in both community and hospital-acquired infections. These results are supported by another study, in which UTIs accounted for about 63% of hospital-acquired infections and 86% were community-acquired (27). Our study also indicated a predominance of women over men (57% vs 43%), when gender-wise distribution was compared; a previous study (27), also reported a higher prevalence of UTIs among females than males (83% vs. 50 %) and *E. coli *was the most frequent uropathogen in both genders. *E. coli *caused 48.29% of urinary tract infections in females and 34.44% in males (9). Other studies also found that women were more likely to have UTIs than men in different geographical regions (28, 29). Regarding the prevalence of UTIs by patients’ gender, our findings are in good agreement with several reports from Pakistan and Bangladesh, which showed that these infections are more common in females as compared with males (6, 9, 30). The higher risk in women is mostly due to the shortness of the female urethra, which is 1.5 inches as compared with 8 inches in men. The prevalence of UTIs was found to be higher in older patients of both genders, whereas, it was low in younger patients having age <20 years. A similar trend has been reported earlier (31), in which UTIs among the elderly, adults, and children were 58.7%, 36.2%, and 5.1%, respectively. The possible causes of the higher incidence of UTIs in the elderly may be due to various factors including urinary tract anomalies, compromised immune response, malnutrition, functional disability, diabetes, and prostate enlargement in males and post-menopausal hormonal changes in females (2, 32). Most commonly prescribed antibiotics to treat urinary tract infections are penicillins (amoxicillin-clavulanic acid), cephalosporins, carbapenems, fluoroquinolones (ciprofloxacin), co-trimoxazole, and aminoglycosides (33).

In the present study, the prevalence of ESBL producers was 65 % in the case of *E. coli *and *K. pneumoniae *similar to another study (18). In contrast, other research (19) has indicated a lower prevalence of ESBL-producing *E. coli *(9 %). Many other reports from different countries and regions showed different prevalence rates of ESBL producing *Enterobacteriaceae *causing urinary tract infections. In a previous study, the prevalence of ESBL producing multidrug-resistant uropathogens *E. coli* and *Klebsiella *sp. was 53% (34). Amoxicillin-clavulanic acid and cephalosporins are the common treatment against ESBL producing uropathogens in treatment centers (35). ESBL-producing *E. coli *and *K. pneumoniae *harbor many ESBL genes on large plasmids, these genes are not only responsible for the higher resistance levels to β-lactam antibiotics but also encode resistance to many other antibiotics including aminoglycosides, quinolones, chloramphenicol, sulfonamides, and tetracycline. ESBL is the presence of a particular beta-lactamase gene that produces an enzyme that is active on a wide spectrum of beta-lactam antibiotics (Amoxicillin, Cefotaxime, Aztreonam, etc.). Usually, the gene is on a plasmid together with many other resistance genes. ESBL works like all other beta-lactamases e.g., breaking the beta-lactam ring (36). Isolated uropathogens (*E. coli *and *Klebsiella*) in this study were highly resistant to ampicillin (87% vs 100%) and least resistant to meropenem (8% vs 21%), respectively. This is similar to the reports of various previous studies (18, 37, 38, 39). However, the isolates exhibited less resistance to chloramphenicol (45% vs 32%), amikacin (35% vs 26%), and nitrofurantoin (25%vs 26%). Also, unexpected higher resistance was detected against amoxicillin-clavulanic acid, ciprofloxacin, cephalosporins, trimethoprim and trimethoprim-sulfamethoxazole, and nalidixic acid. Self-medication, empirical therapy without culture sensitivity testing, and incomplete course of treatment in a community setting may be the major reasons for the development of drug resistance in underdeveloped countries like Pakistan (40). In our results of antimicrobial resistance, the profile is consistent with many previously reported studies (2, 8, 9,37) which indicated that Amikacin, imipenem, and meropenem were highly effective against Gram-negative bacilli causing UTIs and declared the highest resistance to penicillin followed by cephalosporins (first, second, and somewhat third-generation). However, resistance levels to these antimicrobial agents are high enough and clinicians are advised to change these agents in the treatment of urinary tract infections. High-level chloramphenicol resistance in *K. pneumoniae* and *E. coli* is caused by acquisition of the gene Cat (chloramphenicol acetyltransferase), but low-level resistance is caused by mutations that up-regulate drug efflux and mediated by *cml* and *floR* genes. (41). In this study, MIC levels of *E. coli *and *K. pneumoniae *showed a high degree of resistance against trimethoprim, followed by trimethoprim-sulfamethoxazole, ciprofloxacin, and amoxicillin-clavulanic acid. However, a relatively low resistance rate was found against chloramphenicol in these isolates. In contrast, another study (42, 43) has indicated a lower MIC value of ESBL-producing *E. coli *and *K. pneumoniae*. Among 54 FQ-resistant isolates in this study, the most prevalent phylogenetic group was B2 (79.61%) and 20.37% of the isolates belonged to group D. Our results are in accordance with a previous study (44, 45), in which, of the 113 Q- resistant isolates 64.6% belonged to group B2 and 35.4% isolates to the D group. Several previous reports were in contrast to the results of our study, indicating that most of the FQ-resistant isolates belonged to groups B1 and A (46, 47). B2 is a phylogenetic subgroup of *E. coli* that is often resistant to fluoroquinolones. B2 exists and for unknown reasons develops resistance. High level of fluoroquinolone resistance is due to chromosomal mutations causing reduced affinity of DNA gyrase and topoisomerase IV for fluoroquinolones resistance in Gram-negative bacteria is associated with reductions in porins, reduced bacterial accumulation of drug, and overexpression of endogenous MDR pumps. (48, 49). The results of our study showed that most of the FQ-resistant isolates belonged to the B2 group and were PAI*usp *subtype IIa. FQ-resistant isolates carrying resistance mutations in *gyrA *and *parC, *results showed 37 (97.36%) of the 38 isolates of PAI*usp *subtype IIa in phylogenetic group B2 carried E84V mutation in *parC*, while only two of the 11 isolates belonging to group D carried this mutation. In contrast, mutations S80I, S83L, D87N in *parC *and *gyrA *were found in all of the resistant isolates. Our results are consistent with previous studies, showing a high distribution of *parC *E84V mutation present in FQ- resistant isolates of B2 PAI*usp *subtype IIa (50, 51).

**Table 1:** Distribution of isolates belonging to phylogenetic groups B2 and D in PAIusp- subtype

**PAI** ***usp*** ** subtype**	**Phylogenetic group**	
	**B2 (n=43)**	**D (n=11)**
I a	0	0
Ib	0	2
IIa	38	0
IIb	2	0
N.D	3	9

**Table 2:** Genetics of resistance to ciprofloxacin, phylogenetic group, and PAIusp subtype

Phylogenetic group	PAI*usp *subtype	Total no.		Mutation			
				* ParC*		* gyrA*	
	E84V				S80I	S83L	D87N
B2	IIa	38		37	38	38	38
	IIb	2		1	2	2	2
	N.D	3		3	3	3	3
D	Ib	2		0	2	2	2
	N.D	9		2	9	9	9

N.D; PAIusp- negative

## Conclusion

The findings of the present study are alarming in the context of increased resistance among uropathogens to most commonly prescribed antibiotics including penicillin, cephalosporins, and fluoroquinolones. The possible reason for this may be prolonged use of antimicrobial drugs, lax controls on drug availability, self-medication, and prescribing antibiotics without antibiotic susceptibility tests. Moreover, developing countries like Pakistan, Bangladesh, and India have a problem with overuse or misuse of antibiotic agents. Furthermore, there is a strong association between specific patterns of resistance and particular genotypes of the isolates but the relationship is complex. More intensive studies regarding the development of resistance within different phylogenetic groups could be used to predict the general basis for the development of antibiotic resistance. Monitoring, proper screening of multi-drug resistant isolates, and restricted use of antimicrobial agents is necessary and recommended to control high resistance rates of pathogens. 
